# Antimicrobial Resistance of *Serratia marcescens* Recovered From Aquatic Environments: A Review

**DOI:** 10.1111/1758-2229.70345

**Published:** 2026-08-04

**Authors:** Heloisa Silva Inacio, Yasmim Raffaela Soares Santos, Magna Cristina de Paiva, Jaqueline Maria Siqueira Ferreira

**Affiliations:** ^1^ Microbiological Quality Control Laboratory Dona Lindu Midwest Campus, Federal University of São João del Rei São João del Rei Brazil; ^2^ Toxicological Analysis Laboratory Dona Lindu Midwest Campus, Federal University of São João del Rei São João del Rei Brazil; ^3^ Microscopy, Laboratory Diagnosis and Clinical Microbiology Laboratory Dona Lindu Midwest Campus, Federal University of São João del Rei São João del Rei Brazil

## Abstract

*Serratia marcescens* is a gram‐negative bacillus commonly found in aquatic environments. This opportunistic human pathogen can form biofilms and acquire resistance to multiple antimicrobial classes, raising public and environmental health concerns. The occurrence of antimicrobial‐resistant *S. marcescens* in aquatic ecosystems is alarming, yet relatively few studies have addressed this topic in depth. To compile and analyse studies on the antimicrobial susceptibility profiles and resistance mechanisms of *S. marcescens* isolated from aquatic environments. A systematic search was performed in PubMed, Scopus, Web of Science and the Virtual Health Library for articles published between 2019 and 2024, using descriptors related to *S. marcescens* from aquatic sources. Studies evaluating susceptibility profiles of isolates from water samples were included. Thirteen articles met the inclusion criteria, encompassing 90 isolates from diverse water sources. Resistance to β‐lactams was most prevalent, including carbapenems, the most recent compounds in this class. Identified mechanisms included plasmid‐mediated genes blaOXA‐48, blaTEM‐1 and aac(6′)‐Ic. Aquatic environments can serve as reservoirs for resistant *S. marcescens* , posing risks of dissemination to humans and other ecosystems. Continuous monitoring, environmental contamination control and stronger microbiological surveillance policies are crucial to mitigate this emerging public health threat.

## Graphical abstract

*Serratia marcescens* is a gram‐negative bacillus forming biofilms and showing multidrug resistance. Review (2019–2024) included 13 studies, 90 aquatic isolates. High resistance to β‐lactams, including carbapenems. Genes: blaOXA‐48, blaTEM‐1, aac(6′)‐Ic. Aquatic environments are reservoirs, requiring monitoring and control to prevent spread.

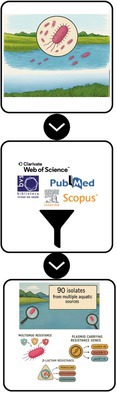

## 1 Introduction

*Serratia marcescens* is a gram‐negative bacillus belonging to the order Enterobacterales and the family Yersiniaceae (Adeolu et al. 2016). It is known for producing prodigiosin, a red pigment that has antimicrobial, antimalarial and antitumor activities (Santos 2020). This facultative anaerobe is mobile, produces catalase and has the ability to adhere to form biofilms on biotic and abiotic surfaces (Hejazi and Falkiner 1997). The natural habitat of *S. marcescens* includes environments such as water, soil and plants and it is part of the intestinal microbiota of homeothermic animals and humans. It has remarkable adaptability: It can grow at a temperature of up to 37°C and tolerate pH between 5 and 9 (Adeolu et al. 2016). According to Tavares‐Carreon et al., there are 23 known *Serratia* species, six of which are associated with human infections: *S. marcescens* , *Serratia plymuthica* , *Serratia liquefaciens* , *Serratia rubidaea* , *Serratia odorifera* and *Serratia fonticola* . Of these, *S. marcescens* is the most prevalent and widely distributed species (Tavares‐Carreon et al. 2023).

*S. marcescens* has often been found in aquatic environments such as surface water, wastewater treatment plants and urban rivers (Mahlen 2011). Studies carried out in China, India and Brazil have identified *S. marcescens* in contaminated waters, where they often present multidrug resistance (MDR) profiles (Inácio et al. 2023; Liu et al. 2023; Soni et al. 2024). Although widely studied in other parts of the world, there are limited data on *S. marcescens* in Brazil and South America and there is a need for a comprehensive survey detailing its distribution in these territories. In Brazil, for example, studies in industrial effluents and urban rivers have shown that this species can present high levels of resistance to antimicrobials and some can be classified as MDR (Inácio et al. 2023; Rave et al. 2018). Continuous exposure to antimicrobial residues in these ecosystems as micropollutants contributes to the selection of resistant strains, representing a challenge for infection control and public health. Thus, these findings reinforce the role of aquatic environments as important reservoirs of resistant bacteria, including *S. marcescens* (Moretto et al. 2021).

*S. marcescens* is an opportunistic pathogen of clinical importance and is associated with serious infections, including of the bloodstream and urinary tract and it can lead to endocarditis (Su et al. 2003). Since the 1990s, outbreaks of *S. marcescens* infections have been documented throughout the world (Woodward and Clarke 1913; Jones 2010). Jones (2010) found *S. marcescens* to be the seventh most common cause of bloodstream infections. Redondo‐Bravo et al. (2019) reported an outbreak in an intensive care unit (ICU) in Spain, highlighting it as the main etiologic agent. In Brazil, there are records of outbreaks related to *S. marcescens* in hospitals, such as the one that occurred in a public ICU in the Federal District in 2020, which resulted in three deaths (Tavares‐Carreon et al. 2023). Moreover, outbreaks in a tertiary hospital in Cuiabá, Mato Grosso and in Duque de Caxias, Rio de Janeiro, were attributed to *S. marcescens* , where it caused serious infections. Furthermore, in São Luís, Maranhão, this species was identified as the cause of endocarditis, with the primary focus of infection being a periodontal abscess (da Cruz et al. 2021; Ribeiro and Aguiar 2010; Silva et al. 2022).

*S. marcescens* is considered to be a growing public health concern due to its ability to develop acquired or adaptive resistance to multiple classes of antimicrobials, in addition to intrinsic resistance to several classes of antimicrobials, such as first‐generation cephalosporins, tetracyclines, polymyxins and nitrofurans (Pitout and Laupland 2008). Intrinsic resistance mechanisms include efflux pumps, modification of outer membrane lipopolysaccharides (LPS) and the production of chromosomal *AmpC* beta‐lactamase. Hence, it is part of the ESCPM group, an acronym that refers to *Enterobacter* spp., *S. marcescens* , *Citrobacter freundii* , *Providencia* spp. and *Morganella morganii* ; this group has high resistance to beta‐lactams (Blanco et al. 2016; Harris and Ferguson 2012; Sandner‐Miranda et al. 2018; Tamma 2024). With regard to acquired resistance, mediated by gene transfer, the production of extended‐spectrum beta‐lactamases (ESBLs) and cephalosporinases stand out, while adaptive resistance occurs in response to environmental stresses, such as exposure to disinfectants or pH changes outside the tolerable range (Begic and Worobec 2008; Maseda et al. 2009).

*S. marcescens* is also highly adept at forming biofilms, which protect bacterial cells from antimicrobials and facilitate the transfer of plasmids carrying antimicrobial resistance genes (Król et al. 2013). In clinical environments, biofilms often contribute to infections related to medical devices, such as catheters. In the aquatic environment, the survival of microbes is associated with the formation of biofilms, which offer protection against adverse conditions and damage caused by various residues from anthropogenic activities and also contribute to the development of antimicrobial resistance (Chen et al. 2020; Ray et al. 2017). The emergence of *S. marcescens* as an MDR pathogen highlights the importance of integrated strategies for monitoring and prevention and the development of new antimicrobial therapies. Given the scarcity of studies detailing the environmental distribution of *S. marcescens* in Brazil and South America and its clinical impact, this review article discusses the available knowledge on this species. We emphasise its aquatic habitat, antimicrobial resistance profile and relevance in the context of public health. This review is especially important from the One Health perspective, which contemplates the exchange of environmental, human and animal bacteria.

## 2 Methodology

This systematic review was carried out in accordance with the *Cochrane Handbook for Systematic Reviews of Interventions* (Higgins and Thomas 2025) and the Preferred Reporting Items for Systematic Reviews and Meta‐Analyses (PRISMA) guidelines (Page et al. 2021).

The PubMed, Scopus, Web of Science and Virtual Health Library (VHL) databases were searched for articles published in English, Portuguese or Spanish between 2019 and 2024. Two researchers (HSI and YRSS) independently conducted the searches from 25 September 2024, to 6 November 2024. A search string based on Medical Subject Heading (MeSH) terms was developed for each database. The search strings were: (‘ *Serratia marcescens* ’) AND (‘waste water’ OR ‘wastewater’ OR ‘sewage’ OR ‘sludge’ OR ‘river’ OR ‘stream’ OR ‘river’ OR ‘streams’ OR ‘Water’) for the PubMed database; ((‘ *Serratia marcescens* ’) AND (‘waste water’ OR ‘wastewater’ OR ‘sewage’ OR ‘sludge’ OR ‘river’ OR ‘stream’ OR ‘river’ OR ‘streams’ OR ‘Water’) OR INDEXTERMS (‘waste water’ OR ‘wastewater’ OR ‘sewage’ OR ‘sludge’ OR ‘river’ OR ‘stream’ OR ‘river’ OR ‘streams’ OR ‘Water’)) for the Scopus database; (‘ *Serratia marcescens* ’) AND (‘water’) for Web of Science; and (‘ *Serratia marcescens* ’) AND (‘wastewater’ OR ‘waste water’ OR ‘Aguas Residuales’ OR ‘water’ OR ‘água’ OR ‘agua’) for VHL.

The general selection criteria for articles after the searches were determined based on the PVS strategy:

- Population: isolates of *S. marcescens* ;
- Variable: antimicrobial resistance profile;
- Study: aquatic environments.

The eligibility criteria included studies that evaluated the antimicrobial resistance profile of *S. marcescens* isolates from aquatic environments. We excluded studies that did not identify the *S. marcescens* species, studies that did not present the antimicrobial susceptibility profile and studies that did not focus on isolates from aquatic environments. In addition, we excluded duplicates between the different databases as well as articles that did not meet the eligibility criteria, non‐original articles, reviews, notes, letters to the editor, abstracts from scientific events and editorials. If a study met the inclusion criteria but the original text was not available, we contacted the corresponding author by email up to three times (with a 14‐day interval between each email). The study was excluded if there was no reply after the third email.

Subsequently, the preselected articles were subjected to a thorough evaluation of the full texts, carried out independently by the researchers, to exclude those that did not meet the proposed eligibility criteria. The following information was extracted from the articles: (i) authors and year of publication; (ii) study site; (iii) type of water sample evaluated; (iv) identification method used; (v) total number of *S. marcescens* isolates; (vi) antimicrobials tested; (vii) number of isolates resistant to each of the antimicrobials tested; (viii) methods used to determine the susceptibility profile; (ix) investigation of resistance mechanisms involved.

The Newcastle‐Ottawa Scale (NOS) (Ribeiro and Aguiar 2010) was used to assess the methodological quality of the cohort studies included in this systematic review. Up to nine stars are awarded to each study, with good‐quality articles receiving five or six stars and excellent‐quality articles receiving seven or more stars.

## 3 Results and Discussion

**FIGURE 1:**
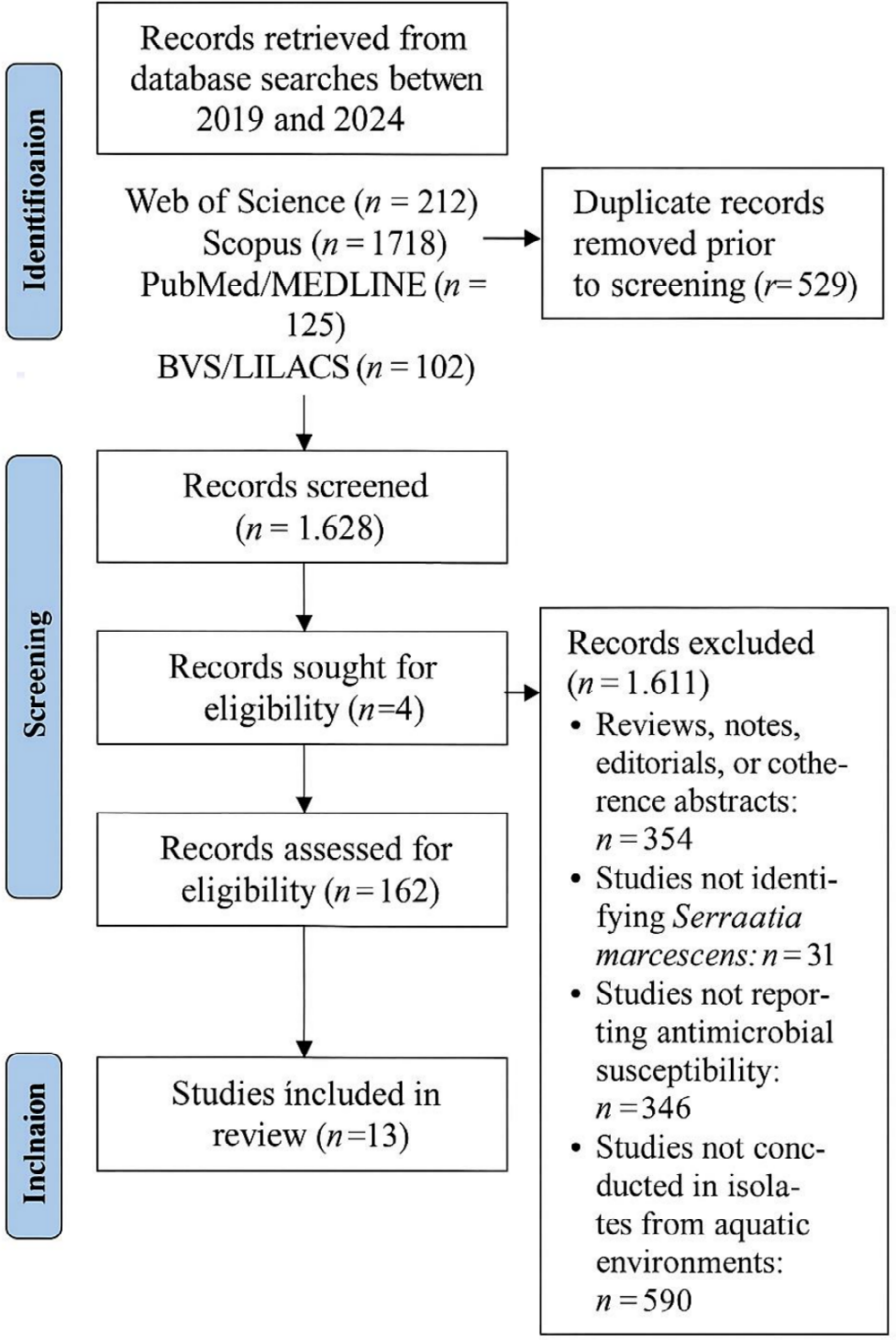
The Preferred Reporting Items for Systematic Reviews and Meta‐Analyses (PRISMA) flowchart for this systematic review.

Our database searches yielded 2157 articles. After applying the eligibility criteria and excluding duplicates, we submitted 17 of them to full‐text reading. In the end, we included 13 of them in this systematic review. Figure 1 presents the PRISMA flow chart of the study selection. Table 1 provides an overview of the 13 included articles.

**TABLE 1:** General characteristics of the studies included in this systematic review.

References	Place of study	Type of sample	Identification method	Total number of isolates	Antimicrobials tested	Total number of isolates resistant to each antimicrobial	Method(s) used to determine the susceptibility profile	Mechanisms/genes of resistance involved
Riedel et al. (2019)	Chesapeake Bay, United States	Water samples from the Chesapeake Bay and its upper tributaries in Maryland	Biochemical‐physiological Mass spectrometry (MALDI‐TOF)	15	Ampicillin, ceftriaxone, ceftaroline, cefepime, piperacillin/tazobactam, imipenem, meropenem, ertapenem, doripenem, ciprofloxacin, gentamicin, tobramycin, chloramphenicol, tetracycline, trimethoprim/sulfamethoxazole	12: ampicillin, 5: chloramphenicol, 6: tetracycline, 3: imipenem, 1: ceftaroline	Disk diffusion, broth microdilution	NF
Moussa et al. (2021)	Orontes River (border of El Qa'a refugee camp) and four other major rivers in Lebanon with possible sewage contamination: Abou Ali River, Awali River, Al Mawt River and Al Berdawni River	River water	Molecular (16S RNA gene sequencing)	1	Amoxicillin, amoxicillin/clavulanic acid, ticarcillin, piperacillin/tazobactam, imipenem, ertapenem, meropenem, aztreonam, cephalothin, cefuroxime, cefoxitin, cefotaxime, ceftriaxone, ceftazidime, cefepime, cefixime, tobramycin, gentamicin, amikacin, ofloxacin, ciprofloxacin, norfloxacin, levofloxacin, tetracycline, tigecycline, trimethoprim/sulfamethoxazole, nitrofurantoin, fosfomycin, colistin	1: amoxicillin, amoxicillin/clavulanic acid, cephalothin, cefuroxime, cefoxitin, tetracycline, nitrofurantoin	Disk diffusion	*bla TEM‐1B*
Shylla et al. (2021)	Khliehriat, in the East Jaintia Hills district of Meghalaya, northeast India	Water from coal mining sites	Biochemical‐physiological Molecular (16S rRNA gene sequencing)	1	Ampicillin, penicillin, streptomycin, tetracycline, nalidixic acid, netilmicin	1: ampicillin and penicillin	Disk diffusion	NI
Tolentino et al. (2021)	São José do Rio Preto, São Paulo, Brazil	Community or individual water wells	Biochemical‐physiological	4	Amoxicillin/clavulanic acid, ampicillin, ampicillin/sulbactam, aztreonam, cefazolin cefepime, cefotaxime cefoxitin, ceftazidime, ertapenem, imipenem meropenem, piperacillin/tazobactam, ticarcillin/clavulanic acid, trimethoprim/sulfamethoxazole, nalidixic acid ciprofloxacin, levofloxacin, amikacin, gentamicin, tobramycin, chloramphenicol, tetracycline	1: chloramphenicol, 4: tetracycline	Disk diffusion	NF
Hassan et al. (2022)	Georgia, United States	Influent sewage and sediment sludge from wastewater treatment plants	Molecular (multilocus ribosomal)	11	Penicillin, ampicillin amoxicillin/clavulanic acid, cefepime, cefotaxime, cefixime, imipenem, doripenem, meropenem, gentamicin, kanamycin, streptomycin, erythromycin, tetracycline, ciprofloxacin, norfloxacin; sulfamethoxazole/trimethoprim, chloramphenicol	11: penicillin, ampicillin, amoxicillin/clavulanic acid, erythromycin, tetracycline	Disk diffusion	*aac(6′)‐Ic bla(SRT‐) (2*), *tet41*
Müller et al. (2022)	Water samples from Lagoa Tramandaí’ (State of Rio Grande do Sul, southern Brazil)	Lagoon water	Mass spectrometry (MALDI‐TOF)	5	Ampicillin, amoxicillin/clavulanic acid, piperacillin/tazobactam, ceftazidime, cefotaxime, cefepime, cefoxitin, imipenem, tetracycline, amikacin, gentamicin, chloramphenicol, norfloxacin, aztreonam, nitrofurantoin, sulfamethoxazole/trimethroprim	11: amoxicillin/clavulanic acid, ampicillin, 5: cefoxitin, 4: chloramphenicol, 8: nitrofurantoin, 2: sulfamethoxazole/trimeth oprim, 1: gentamicin, 15: tetracycline	Disk diffusion	*blaOXA‐48*
Nakamura‐Silva et al. (2022)	Secondary hospital in Ribeirão Preto, southeastern Brazil	Sewage	Mass spectrometry (MALDI‐TOF)	4	Ciprofloxacin, levofloxacin, lomefloxacin, ofloxacin, norfloxacin, nalidixic acid, amikacin, streptomycin, gentamicin, tobramycin, netilmicin, doripenem, ertapenem, imipenem, meropenem, cefazolin, cefaclor, cefotetan, cefoxitin, cefuroxime, ceftazidime, cefixime, ceftriaxone, cefotaxime, cefepime, ceftaroline, aztreonam, amoxicillin/clavulanate, ampicillin/sulbactam, piperacillin/tazobactam, ticarcillin/clavulanate, doxycycline, minocycline tetracycline, nitrofurantoin, sulfonamides, trimethoprim/sulfamethoxazole, chloramphenicol and fosfomycin	1: ceftazidime, ceftriaxone, ticarcillin/clavulanate, minocycline, trimethoprim/sulfametho xazole, meropenem, 2: tobramycin, ceftaroline, chloramphenicol, tetracycline, 4: streptomycin, doxycycline, nitrofurantoin, sulfonamides, piperacillin/tazobactam, 3: doripenem, cefixime, cefotaxime, ceftaroline	Disk diffusion	NF
El‐Sesy and Othman (2023)	Sharqia, Menufia and Port Saied provinces, Egypt	Agricultural drainage water, industrial wastewater and water contaminated with sewage	Biochemical‐physiological	1	Amoxicillin/clavulanate, ampicillin, vancomycin, neomycin, tetracycline, clindamycin, erythromycin, cefotaxime, cefepime, ofloxacin	Amoxicillin/clavulanate, ampicillin, ofloxacin, tetracycline, neomycin, erythromycin, cefotaxime, cefepime and vancomycin	Disk diffusion	NF
Inácio et al. (2023)	Divinópolis‐MG, Brazil	Urban river water	Biochemical‐physiological	19	Fosfomycin, aztreonam, ciprofloxacin, amikacin, gentamicin, ceftazidime, cefepime, ceftriaxone, cefoxitin, meropenem, ertapenem, imipenem, cefotaxime, sultamethoxazole‐trimethoprim and chloramphenicol	1: ciprofloxacin, amikacin, 2: ceftazidime, cefepime, ertapenem, ceftriaxone, 3: aztreonam, gentamucine, 8: chloramphenicol, cefoxitin, 10: Fosfomycin, 11: sultamethoxazole‐ trimethoprim	Disk diffusion	NI
Liu et al. (2023)	Qishan River, from near its source at the foot of Yushan Mountain to its confluence with the Kaoping River, Taiwan, China	River water	Biochemical‐physiological	4	Ampicillin, cefotaxime chloramphenicol, ciprofloxacin, erythromycin, gentamicin, tetracycline, trimethoprim, trimethoprim/sulfamethoxazole and vancomycin	None	Disk diffusion, broth microdilution	NF
Pasoń and Stańczyk‐Mazanek (2023)	Municipal wastewater treatment plant in a city with a population of over 30,000 residents	Raw sewage sludge	Biochemical‐physiological	1	Ampicillin, amoxicillin/clavulanic acid, amikacin, gentamicin, cefuroxime, cefazolin, ceftazidime and ciprofloxacin.	None	Disk diffusion	NF
Suresh et al. (2023)	Krishna and West Godavari of Andhra Pradesh, India.	Water from fish farms	Biochemical‐physiological	13	Furazolidone, oxytetracycline, doxycycline hydrochloride, cotrimoxazole, enrofloxacin and ciprofloxacin	4: furazolidone, 13: oxytetracycline and doxycycline hydrochloride	Disk diffusion Broth microdilution	NF
Kanarek et al. (2024)	Kuyavian‐Pomeranian and Greater Poland Voivodeships, Poland	Vegetable and fruit washing water	Mass spectrometry (MALDI‐TOF)	1	Piperacillin, ceftazidime cefiderocol, imipenem meropenem, tobramycin, levofloxacin, ciprofloxacin, ticarcillin/clavulanic acid, cefepime, amikacin, gentamicin, moxifloxacin, amoxicillin/clavulanic acid, trimethoprim, ampicillin, tigecycline, linezolid, vancomycin	None	Disk diffusion	NF

Abbreviations: MALDI‐TOF, matrix‐assisted laser desorption/ionisation time of flight; NF, not found; NI, not investigated.

*S. marcescens* plays a significant role in the clinical setting, especially as a nosocomial pathogen associated with serious infections in immunocompromised patients. Its presence in healthcare environments, combined with its ability to survive on surfaces and its antimicrobial resistance, makes its spread a constant concern (Tavares‐Carreon et al. 2023). In addition, evidence suggests there is exchange between environmental and clinical reservoirs of this species. This phenomenon reinforces the need for an integrated One Health approach between the environment, humans and hospital infrastructure to understand the ecology and evolution of *S. marcescens* (Bourdin et al. 2023).

Despite the relevance of this topic, we only identified 13 articles that have investigated the antimicrobial susceptibility profile in *S. marcescens* isolates recovered from the aquatic environment. The scarcity of research investigating the relationship between environmental and clinical isolates of *S. marcescens* limits understanding of the spread and evolution of resistance in this pathogen, making it difficult to implement effective control and prevention strategies; so we sought to expand knowledge and to encourage further research in this context.

Of the 13 included articles, four were carried out in Brazil (two in the state of São Paulo, one in Rio Grande do Sul and one in Minas Gerais). The others were performed in India, Poland and the United States (two each), as well as China, Lebanon and Egypt (one each). Although there has been relatively little research in this area, Brazil is leading the way in research involving *S. marcescens* in aquatic environments. Of note, there has been an increase in the number of publications in this area since 2023, which suggests greater concern about the aquatic spread of *S. marcescens* . This increase in research is also probably due to an increased interest in wastewater epidemiology. This concept has been around since mid‐2001, but has only become popular since the coronavirus disease 2019 (COVID‐19) pandemic. In essence, this concept revolves around the tracking of biomarkers in sewage and/or wastewater samples, which brings significant advances to public health because it promotes tools that assist in decision‐making, implementation of interventions, prevention, control and treatment of diseases (Afonso Cabral et al. 2024).

There is an increasing need for research into the emergence of MDR bacterial strains in aquatic environments. Huijbers et al. (2020) and Hutinel et al. (2019) have used samples extracted from sewage to produce relevant insights into the prevalence of *Escherichia coli* and its genetic components linked to antimicrobial resistance, which have not been observed for *S. marcescens* .

In the included articles, different types of surface water, such as river water, well water, food washing water and sewage, among others, have been investigated for the presence of *S. marcescens* (Hassan et al. 2022; Müller et al. 2022; Nakamura‐Silva et al. 2022; El‐Sesy and Othman 2023; Pasoń and Stańczyk‐Mazanek 2023). Considering the particularities of each sample, this diversity of samples did not allow us to relate the findings of this species to the specific characteristics of each sample type. Hence, there is a need to strengthen research in this area. The larger number of studies that evaluated sewage samples can be explained by the high concentration of pathogenic microorganisms found in them. Microbial loads derived from domestic, industrial and hospital waste are often related to the exchange of resistance genes, making them a strategic point for epidemiological health surveillance. Mao et al. (2020) noted that sewage monitoring has been widely used as an efficient tool for the detection of emerging pathogens, reinforcing its importance in environmental and public health studies. In this context, the inadequate disposal of hospital waste stands out. It contains microorganisms resistant to multiple classes of antimicrobials as well as drug residues that can contribute to the selection of resistant microorganisms. Rizzo et al. (2013) showed that hospital effluents are significant sources of resistance genes and opportunistic pathogens, such as *S. marcescens* , which can persist in the environment and be redistributed to other ecological compartments.

Another type of sample evaluated by the studies was sludge from wastewater treatment plants, which also represents the aquatic environment and is related to biological water treatment processes (Hassan et al. 2022; Müller et al. 2022; Nakamura‐Silva et al. 2022; El‐Sesy and Othman 2023; Pasoń and Stańczyk‐Mazanek 2023). Hassan et al. (2022) found MDR *S. marcescens* in this type of sample. This finding is certainly due to the fact that antimicrobial‐resistant bacteria and drug residues arrive at wastewater treatment plants along with the water, but are not removed by the treatments. Therefore, they remain in the sludge and the treated effluent that reaches the waterways, posing risks to human and animal health and facilitating the emergence of antimicrobial resistance due to the selective pressure they exert on the local microbial community. Due to health risks, sewage sludge after wastewater treatment requires additional management, which includes steps such as biological or chemical stabilisation, dewatering and sanitisation by thermal or chemical methods to reduce the pathogenic load and the presence of emerging contaminants such as antimicrobial resistance genes. These measures are essential to guarantee environmental and health safety, especially when the treated sludge is destined for agriculture or other forms of reuse (An et al. 2018). Nevertheless, these complementary treatments are not always implemented, mainly due to economic, technical and regulatory barriers. Recent studies have highlighted that high operating costs, the need for specialised infrastructure and the lack of specific and standardised regulations in many countries hinder the systematic application of advanced sludge treatment technologies (Hua 2025; Zhang et al. 2018).

Still considering health risks, the findings reported by Suresh et al. (2023) and Kanarek et al. (2024) stand out; both are related to food risks. Suresh et al. (2023) reported the presence of MDR *S. marcescens* in water samples from fish farms. Although this bacterium is directly associated with fish, these MDR strains can play an important role in the spread of antimicrobial resistance through horizontal interspecies gene transfer in the aquatic environment. Fish farming relies heavily on the use of antimicrobials to combat infectious diseases and as growth promoters for animals. However, the excessive use of these compounds poses risks to food and environmental safety because these strains remain viable even after disinfection processes and should therefore be monitored and their use reviewed (Gadhiya et al. 2021).

Kanarek et al. (2024) evaluated samples of water used for washing and processing vegetables and fruits intended for human consumption and reported the risk of cross‐contamination if the water used is not exchanged and/or disinfected between batches. Disinfection is one of the most important steps and if not carried out correctly, it affects the quality and safety of the product intended for human consumption. In these processes, the washing water can serve as a habitat for microorganisms, especially if biofilm is formed on the inside walls of the tanks and pipes. According to Gu et al. (2021), the main point of bacterial contamination is during the post‐harvest stage, where the presence of residues and vegetables in the water promotes bacterial multiplication and adhesion to the surfaces of vegetables and fruits.

Together, the articles included in this systematic review evaluated 90 *S. marcescens* isolates from aquatic environments. This is a relatively small number, especially given that the aquatic environment is the main habitat of *S. marcescens* . The study by Inácio et al. (2023) carried out in Brazil stands out, as it included the largest number of isolates (*n* = 19) of the 13 included articles. The limited number of isolates in the studies can be attributed to various factors, including the difficulty in recovering environmental isolates due to the complexity and diversity of the local microbiota, making their detection and isolation more challenging (Carlsen et al. 2022). In fact, when compared to studies from the clinical setting, the number of environmental isolates observed here is low. For example, Ferreira et al. (2020) obtained 54 *S. marcescens* isolates from clinical samples, Rodrigues et al. (2020) reported 30 *S. marcescens* isolates during a single outbreak in a referral hospital in Belém, Brazil and Hanczvikkel et al. (2024) found 18 *S. marcescens* isolates during an outbreak in an adult ICU in Hungary.

With regard to microbiological diagnostic methods, the most commonly used to identify *S. marcescens* were biochemical‐physiological (8 of the 13 included articles). Molecular methods (3 of the 13 included articles) and mass spectrometry, specifically matrix‐assisted laser desorption/ionisation time of flight (MALDI‐TOF) (4 of the 13 included articles) were also used and the authors of two studies combined these two methods. Specifically, Riedel et al. (2019) used biochemical‐physiological tests and mass spectrometry and Shylla et al. (2021) combined biochemical‐physiological and molecular tests. Specifically for the studies carried out in Brazil, the methods used were biochemical‐physiological (1 of the 3 included articles) and mass spectrometry (2 of the 3 included articles). In Brazil, mass spectrometry has been available since 2004, but it only began to be widely used in 2009. Thus, there has been an evolution of the methodological quality in Brazilian research (Tsuchida et al. 2020; Qiao 2022). Molecular methods such as polymerase chain reaction (PCR) and advanced techniques such as mass spectrometry offer greater robustness for bacterial identification. These approaches have superior sensitivity and specificity compared with traditional biochemical‐physiological methods, allowing for faster and more accurate identification of bacteria. For this reason, their adoption is recommended in clinical and research laboratories, especially in contexts that require agile diagnostic responses (Kunduracılar 2019).

Antimicrobial susceptibility tests are of interest and relevance beyond the clinical context where they guide antimicrobial therapy. The definition of the sensitivity profile in extra‐clinical isolates is interesting from an epidemiological point of view, especially given the possibility of bacterial exchange between these environments. Among the included articles, the disk‐diffusion method was the most commonly used for this purpose. This qualitative method categorises bacteria as sensitive, sensitive by increasing the dose (intermediate) and resistant according to the Clinical and Laboratory Standards Institute (CLSI), European Committee on Antimicrobial Susceptibility Testing (EUCAST) and Brazilian Committee on Antimicrobial Susceptibility Testing (BRCAST) reference protocols (BrCAST 2024; eucast: Resistance Mechanisms 2025; Clinical and Laboratory Standards Institute 2024). It is widely used in clinical microbiology because it is more cost‐effective and contributes to the choice of effective antimicrobials (Balouiri et al. 2016). However, the broth microdilution method is considered to be the gold standard, enabling the determination of the minimum inhibitory concentration (MIC), which is important for guiding the most effective therapeutic choice and monitoring the evolution of antimicrobial resistance (Campana et al. 2011). In addition, the result obtained by using this method is fundamental for dose adjustment, as it allows the therapeutic regimen to be customised, helping to avoid both treatment failures and toxicity associated with underdosing or overdosing (Mouton et al. 2018).

It is worth mentioning that the disk diffusion and microdilution methods have been used by some researchers and discordant results have been observed for some antimicrobials. Liu et al. (2023) applied these methods to the same antimicrobials and observed discrepancies in the results related to the profile of ampicillin and chloramphenicol, with a change in the classification of the isolate from sensitive to intermediate. On the other hand, Riedel et al. (2019) and Suresh et al. (2023) used both methods to determine the antimicrobial susceptibility profile and found no difference in the results between the methods. It is important to note that, considering the methodological robustness of broth microdilution, its result should define the categorisation of the antimicrobial for the bacterium studied.

*S. marcescens* is known to have developed resistance to various antimicrobials of clinical relevance. In the included articles, the susceptibility profile to antimicrobials from various classes was determined, including beta‐lactams, cephalosporins, carbapenems, monobactams, aminoglycosides, macrolides, lincosamides, tetracyclines, fluoroquinolones, and sulphonamides (Table 1).

As previously mentioned, *S. marcescens* is intrinsically resistant to first‐generation cephalosporins (cefazolin, cephalothin and cefadroxil), minocycline, tetracycline, doxycycline, colistin and nitrofurantoin, so susceptibility profile tests for these antimicrobials are not relevant (Tavares‐Carreon et al. 2023). Nevertheless, 9 of the 13 included articles examined tetracycline in the susceptibility tests; 3 included nitrofurantoin; 2 included cefazolin; and 1 evaluated doxycycline, cephalothin, minocycline and colistin. Many of these articles reported sensitivity to these antimicrobials. This contradiction can be explained by variations in the expression of resistance mechanisms and specific characteristics of certain strains, as well as possible flaws in the methodology used (Zhang et al. 2018). In addition, the authors used the disk‐diffusion method, whose results are affected by the bacterial inoculum: A concentration that is too high can lead to false resistance or sensitivity. In addition, the use of unsuitable culture media compromises the diffusion and activity of antimicrobials, while incubation temperatures and atmospheres outside the recommended parameters can alter bacterial growth. Finally, incorrect reading and interpretation of the diameter of the inhibition zones can lead to incorrect categorisation of susceptibility. Therefore, this test must be subjected to quality control to guarantee the fidelity of the results. Moreover, there must be rigorous selection of the antimicrobials that are included, always obeying the criteria of the reference protocols adopted in the country of origin of the study (Reinert et al. 2007; Reller et al. 2009). Of the 13 included articles, 9 evaluated the susceptibility profile of *S. marcescens* using the criteria established by the CLSI. Two articles followed the EUCAST protocol and one article used the BRCAST protocol. These protocols include antimicrobials recommended for susceptibility testing of gram‐negative bacteria, including drugs widely used in clinical practice for the treatment of infections caused by species of the order Enterobacterales.

With regard to the antimicrobial resistance of *S. marcescens* , there was variation observed in the susceptibility profile to beta‐lactams to which this species does not show intrinsic resistance (aztreonam, piperacillin, amoxicillin, amoxicillin/clavulanic acid, ampicillin, cefuroxime, ceftriaxone, ceftazidime, cefepime, ceftaroline, ertapenem, doripenem and meropenem). Among the isolates evaluated, there was greater sensitivity to second‐, third‐ and fourth‐generation cephalosporins (52/90, 57.7%). However, the finding of isolates resistant to these compounds (Inácio et al. 2023; Moussa et al. 2021; Müller et al. 2022; Nakamura‐Silva et al. 2022; El‐Sesy and Othman 2023) can be explained by the fact that *S. marcescens* belongs to the ESCPM group, which shares the *AmpC* gene in a natural and chromosomal form. This gene is overexpressed upon exposure to beta‐lactam antimicrobials (Stewart et al. 2021). In addition, some isolates were resistant to penicillins (piperacillin, amoxicillin, amoxicillin/clavulanic acid and ampicillin: 28/90, 31.1%) and monobactams (aztreonam: 3/90, 3.3%). These data indicate that, despite the known intrinsic resistance of *S. marcescens* to some antimicrobials, there are still effective therapeutic options within the beta‐lactam class.

The production of chromosomal *AmpC* beta‐lactamase in *S. marcescens* confers natural resistance to penicillins and first‐generation cephalosporins (Tavares‐Carreon et al. 2023). The presence of plasmid *AmpC* is less frequent in this species, although it can occur, especially in isolates from hospital environments. In a clinical context, Peter‐Getzlaff et al. (2011) observed that *AmpC* production in this species is generally associated with the chromosomal gene. Similarly, an analysis carried out between 2018 and 2021 indicated that the presence of plasmid *AmpC* in *S. marcescens* is relatively rare compared with other Enterobacterales (Rodríguez‐Guerrero et al. 2022). In environmental isolates, it is also uncommon for isolates to carry plasmid *AmpC*, as shown in an analysis of isolates from river water in Switzerland, in which this gene was not detected in *S. marcescens* (Zurfluh et al. 2015).

Still, among the beta‐lactams, the inclusion of carbapenem antimicrobials (meropenem, imipenem, ertapenem and doripenem) in the majority of the included articles (10/13) should be highlighted (Inácio et al. 2023; Liu et al. 2023; Riedel et al. 2019; Moussa et al. 2021; Tolentino et al. 2021; Hassan et al. 2022; Müller et al. 2022; Nakamura‐Silva et al. 2022; Kanarek et al. 2024). Considering the 90 isolates in the 13 included articles, for carbapenems there was a sensitivity rate of 41.1% (37/90) and a resistance rate of 10% (9/90). These compounds represent the most recent generation of beta‐lactams available in clinical practice and are especially indicated for the treatment of serious infections caused by MDR bacteria (Papp‐Wallace et al. 2011). In clinical isolates, resistance of this species to carbapenems has frequently been reported (Jia et al. 2023; Das et al. 2023; do Prado et al. 2021). It should be remembered that bacteria from the order Enterobacterales, which includes *S. marcescens* , with resistance to carbapenems are listed as a critical priority by the World Health Organization for monitoring the spread of resistance and developing new drugs. Given the finding of *S. marcescens* with this phenotype in water samples, it is imperative to expand research with this approach to define the context of its distribution and to develop control strategies (WHO 2024).

There was an inconsistency observed in some studies that was the result of the susceptibility test to antimicrobials such as vancomycin, erythromycin, clindamycin, streptomycin and furazolidone (Table 1), which are not aimed at gram‐negative bacteria and do not have breakpoints defined by the reference protocols for antimicrobial susceptibility tests. For example, erythromycin and vancomycin act against gram‐positive bacteria (Selim 2022), so the resistance observed does not necessarily reflect a relevant impact on the profile of *S. marcescens* , considering that these antimicrobials are neither prescribed for nor effective in treating infections caused by it.

The mechanisms of antimicrobial resistance in *S. marcescens* isolates were investigated in only 7 of the 13 included articles and only 3 of the articles detected antimicrobial resistance genes (Table 1). Hassan et al. (2022) found three antimicrobial resistance genes (*aac(6′)‐Ic*, *blaSRT‐2* and *tet41*) that confer resistance to aminoglycosides, beta‐lactams and tetracycline, respectively (Król et al. 2013). These findings may justify the resistance profile to some antimicrobials found by these authors, including isolates resistant to penicillin, ampicillin, amoxicillin/clavulanic acid and tetracycline. Müller et al. (2022) detected the *blaOXA‐48* gene in two isolates, which confers resistance to carbapenems, penicillins and monobactams (Santajit et al. 2024). However, in that study, the isolates showed sensitivity to carbapenems, which initially seems contradictory in relation to the presence of the *bla*_*(OXA‐48*)_ gene. This discrepancy may be related to low or absent expression of these genes, mutations in regulatory regions, or the presence of associated resistance mechanisms such as loss of porins or overexpression of repressed efflux pumps, which do not manifest themselves phenotypically (Santajit et al. 2024; Pedraza et al. 2022). Moussa et al. (2021) detected the presence of the *bla*_TEM‐1_ gene, which confers resistance to penicillins (such as ampicillin and ticarcillin) and some first‐generation cephalosporins, in their studied isolate. This gene encodes a TEM‐type plasmid beta‐lactamase, historically considered to be one of the first ESBLs described (Sandner‐Miranda et al. 2018). This finding is consistent with the resistance profile observed in the isolate, especially associated with the chromosomal betalactamase *AmpC* of *S. marcescens* , which contributes to resistance to cefoxitin and cefuroxime (Tebano et al. 2024).

Four of the included articles (Inácio et al. 2023; Moussa et al. 2021; Hassan et al. 2022; Nakamura‐Silva et al. 2022) detected MDR *S. marcescens* , meaning with resistance to at least one antimicrobial from three or more different classes, in the aquatic environment (Magiorakos et al. 2012). This resistance to various classes of antimicrobials has increased among bacterial isolates, causing great concern in the medical community, as it makes it difficult to treat infections caused by MDR bacteria (Valiatti et al. 2023). The identification of MDR *S. marcescens* in extra‐clinical environments has become increasingly frequent and several studies have reported the presence of MDR strains in different non‐hospital sources, such as treatment plant water, dog wounds and plants, indicating that the environment even acts as an important reservoir of antimicrobial resistance (Soni et al. 2024; Machado et al. 2022; Maina et al. 2021).

Finally, when correlating the antimicrobial resistance profile with the origin of the isolates, samples from lakes, sewers and urban rivers showed the highest levels of resistance to the different classes of antimicrobials tested, reinforcing the role of these environments as important reservoirs of resistant bacteria (Inácio et al. 2023; Müller et al. 2022; Nakamura‐Silva et al. 2022; Silva et al. 2024). These environments are more exposed to discharges of domestic, hospital and industrial effluents, which carry anthropogenic waste that favours the selection and dissemination of resistant microorganisms (Evoung Chandja et al. 2024). In fact, aquatic environments are recognised as hotspots of antimicrobial resistance. Batista et al. pointed out that hospital sewage acts as an important disseminator of bacteria and resistance genes, while Dropa et al. showed that, even after conventional treatment, effluents from human hospitals, veterinary hospitals and sewage treatment plants still had resistance genes (Batista et al. 2022; Dropa et al. 2024). Marques et al. (2024) observed that water and sediment samples from urban rivers contained antimicrobial‐resistant bacteria and genes associated with resistance and warned of the possibility of this scenario worsening with an increase in antimicrobial resistance.

We used the NOS, based on criteria relating to selection and comparability between cohorts and outcomes, to assess the quality of the included articles (Table 2) (Ribeiro and Aguiar 2010). One article received nine stars and two articles received eight stars, so they were of excellent quality. In addition, four articles received seven stars and were therefore of good quality. These results indicate that the review is based on solid evidence, making the conclusions more reliable. The predominance of studies with high scores reinforces the methodological robustness of the analysis carried out, reducing the risk of bias and increasing the external validity of the findings.

**TABLE 2:** Assessment of study quality according to the Newcastle‐Ottawa Scale.

References	Selection			Comparability	Outcome	Total points
	1	2	3	4 5	6 7 8	
El‐Sesy and Othman (2023)	*	*	—	— *	* * —	5
Hassan et al. (2022)	*	—	—	— —	* * *	4
Inácio et al. (2023)	*	*	*	— **	* * *	8
Kanarek et al. (2024)	*	*	—	— **	* * *	7
Liu et al. (2023)	*	*	—	— ***	** * *	9
Moussa et al. (2021)	*	*	—	— —	* — —	3
Müller et al. (2022)	*	*	—	* *	* * *	7
Nakamura‐Silva et al. (2022)	*	*	*	— *	* * *	7
Pasoń and Stańczyk‐Mazanek (2023)	*	—	*	— *	* * *	6
Riedel et al. (2019)	*	—	—	— *	** * —	5
Shylla et al. (2021)	—	*	—	— —	* * *	4
Suresh et al. (2023)	*	*	*	* *	* * *	8
Tolentino et al. (2021)	*	*	*	— *	* * *	7

*Note:* Received a star, as the exposed cohort was somewhat representative of the average in the community. 2—Selection of the unexposed cohort: All studies received a star, as the unexposed cohort was obtained from the same community as the exposed cohort. 3—Determination of exposure: Only studies that used the microdilution method to detect carbapenem resistance received a star. 4—Demonstration that the outcome of interest was not present at the start of the study: Not applicable (NA). 5—Comparability of the cohort based on design and analysis: Studies that assessed the presence of infection caused by carbapenem‐resistant gram‐negative bacteria separately in patients with chronic kidney disease and undergoing dialysis or kidney transplant patients and in which the patients had a urinary tract infection received 2 stars. 6—Determination of the outcome: All studies received one star because the assessment of the outcome was carried out in a blinded and independent manner. 7—Sufficient follow‐up period for the occurrence of the outcome: Studies in which patients were followed up for at least 6 months received one star. 8—Adequacy of the cohort follow‐up period: Studies in which all patients were followed up until the end, making it possible to verify the presence or absence of bacterial resistance, received one star.

Among the limitations of this study is the small number of articles included that isolated *S. marcescens* from the aquatic environment and analysed its susceptibility profile to antimicrobials. Moreover, the included articles used less robust methodologies. Therefore, this systematic review demonstrates the need for more in‐depth studies on the subject, as well as the implementation of public policies aimed at the environment in particular. Despite these limitations, this review has provided relevant and worrying information, especially for Brazil, where the rates of human infections by resistant *S. marcescens* have been gradually increasing and the finding in aquatic environments is evidence of yet another source of contamination by this species. Furthermore, *S. marcescens* presents intrinsic resistance to various antimicrobials and due to its great plasticity, it can easily acquire resistance to various classes of antimicrobials. This represents a challenge for the scientific community and a potential public health problem.

## 4 Conclusion

*S. marcescens* from aquatic environments is an important reservoir of antimicrobial resistance, exhibiting diverse resistance mechanisms and the potential for interspecies dissemination. The detection of MDR isolates in surface water and effluents highlights the need for more in‐depth study to understand the context of antimicrobial resistance in these environments. Further research in this area is essential to support public policies aimed at microbiological surveillance to control the spread of antimicrobial‐resistant *S. marcescens* of clinical relevance.

## Author Contributions

**Heloisa Silva Inacio:** investigation, writing – review and editing, visualization, formal analysis, data curation, validation. **Magna Cristina de Paiva:** writing – original draft, writing – review and editing, visualization, methodology, project administration, supervision. **Yasmim Raffaela Soares Santos:** investigation, visualization, writing – review and editing, validation, formal analysis, data curation. **Jaqueline Maria Siqueira Ferreira:** funding acquisition, visualization, formal analysis, resources.

## Funding

This work was supported by the Coordenação de Aperfeiçoamento de Pessoal de Nível Superior (001).

## Conflicts of Interest

The authors declare no conflicts of interest.

## Data Availability

Data sharing not applicable to this article as no datasets were generated or analysed during the current study.
